# Availability of Treatment for Eclampsia in Public Health Institutions in Maharashtra, India

**DOI:** 10.3329/jhpn.v31i1.14753

**Published:** 2013-03

**Authors:** Sarika Chaturvedi, Bharat Randive, Nerges Mistry

**Affiliations:** Foundation for Research in Community Health, Pune, India

**Keywords:** Anticonvulsant eclampsia, Eclampsia epidemiology, Eclampsia therapy, Magnesium sulphate, Pregnancy complication, Public health system, India

## Abstract

Severe pre-eclampsia and eclampsia are common causes of maternal deaths worldwide and more so in developing countries. Magnesium sulphate (MgSO_4_) is now the most-recommended drug of choice to treat these conditions. Despite favourable policies for the use of MgSO_4_ treatment in India, eclampsia continues to take a high toll. This study examined the availability and use of MgSO_4_ treatment in the public health system and poor women's recent experiences with eclampsia treatment in Maharashtra state. A mix of qualitative and quantative methods was used. A facility-based survey of all secondary and tertiary healthcare facilities (n=44) in 3 selected districts and interviews with public and contracted-in private sector obstetricians, health officials, and programme managers were conducted. A list of recently-delivering women from marginalized communities, with up to two livebirths, was drawn through a community-level survey in 272 villages covered by 60 subcentres selected at random. Mothers were selected for interviews, using maximum variation sampling, and interviews were conducted with 17% of the mothers who reported having experienced eclampsia; 61% of facilities had no stock of MgSO_4_, the stock-out position continuing from a period ranging from 3 months to 3 years while another 20% had some stock, although less than the expected minimum quantity. No treatment for eclampsia was provided in the recent 3 months at 73% facilities. Our survey of recently-delivering mothers recorded a history of eclampsia in 3.2% pregnancies/deliveries. Interviews with 10 such mothers revealed that treatment for eclampsia has been sought from public as well as private hospitals and from traditional healers. However, facilities where women have received medical treatment are exclusively in the private sector. Almost all public and private care providers were aware of MgSO_4_ as the gold standard to treat eclampsia; however, it is unclear if they knew of its use to treat severe pre-eclampsia. The private care providers routinely used MgSO_4_ for eclampsia treatment while the public care providers seemed hesitant to use it fearing risks of complications. We stress the need for improved inventory control practices to ensure sustained availability of supplies and building confidence of care providers in using MgSO_4_ treatment for severe pre-eclampsia and eclampsia in public facilities, in addition to teaching expectant mothers how to recognize symptoms of these conditions.

## INTRODUCTION

Pregnancy and childbirth are a reason to celebrate but for half a million women worldwide childbirth turns fatal every year. Almost all these deaths occur in the developing world (1), with India contributing a quarter of these deaths (2). Pre-eclampsia, the rapid elevation of blood pressure during pregnancy, if untreated, can lead to seizures (eclampsia), kidney and liver damage and, ultimately, death. Pre-eclampsia/eclampsia ranks second only to haemorrhage as a specific direct cause of maternal mortality; it caused 63,000 deaths worldwide in 2002 (3). Pre-eclampsia/eclampsia is associated with four times higher risk of newborn deaths. The World Health Organization (WHO) estimates the risk of dying due to pre-eclampsia/eclampsia as approximately 300 times higher for a woman in a developing country than for one in a developed country (4). Unfortunately, pre-eclampsia is not preventable, nor is its onset accurately predictable.

Large multicentre randomized controlled trials (5,6,7) and systematic reviews (8,9,10) provide strong evidence of the effectiveness of magnesium sulphate (MgSO_4_) treatment for severe pre-eclampsia and eclampsia. However, this evidence has not necessarily been translated into policy and/or practice internationally (11,12). The WHO guidelines recommend MgSO_4_ as an essential drug (13) and as the most effective, safe, and low-cost drug to treat severe pre-eclampsia and eclampsia (14) but only about half of the countries in the world follow these WHO guidelines.

The maternal mortality ratio (MMR) for India in 2009 was 212 per 100,000 livebirths (15), with hypertensive disorders causing 5% of maternal deaths (16). India includes MgSO_4_ in its national list of essential medicines (17). Therefore, MgSO_4_ is to be available in a functioning health system at all times in adequate amounts, with assured quality. Its administration is included in the package of basic emergency obstetric care (EmOC) services supposed to be available at all Community Health Centres (CHCs) in India. Despite these policies, many women continue to succumb to eclampsia. This condition emerged as the number one killer in Maharashtra state of India where it contributed to a startling 26% of the maternal deaths (18). There remains a dearth of data that could explain the gaps in services that create this scenario. This paper examines issues relating to the availability of magnesium sulphate treatment for eclampsia in the public health system in Maharashtra and poor women's recent experiences with eclampsia treatment.

## MATERIALS AND METHODS

### Study design

This is a community- and hospital-based cross-sectional study. We used a mix of quantitative and qualitative methods to examine both provision and use of specific services with regard to medical treatment for eclampsia. We conducted a community-level survey of recently-delivering women and a survey of public health facilities during 2009-2010.

### Study settings

Maharashtra state, with a population of nearly 97 million, has 35 districts comprising 358 blocks and 43,711 villages. Compared to national averages, Maharashtra has a higher level of economic growth and, in terms of the Human Development Index (HDI), the state ranked eleventh in 2006 with an HDI of 0.689 (19). The MMR for Maharashtra is 104, and the infant mortality rate is 31 (15). The public health system of the state has a network of 2 super-specialty hospitals in non-metropolitan areas and 23 district hospitals, along with 8 women's hospitals and 446 rural/subdistrict hospitals. The latter ones are secondary-care centres; rural hospitals are also called Community Health Centres (CHCs) with specialty services and are located at the block level catering to about 80,000–120,000 population.

### Sampling and data collection

We used multistage and non-probability sampling to select districts. Villages were selected randomly for community-level survey. Public health staff members were automatically selected from the study facilities for interview. Women for interview were selected based on maximum variation sampling.

Three districts, representing the state's geographic regions, were selected on the basis of heterogeneity in the sociodemographic profile as reflected from HDI 2002 (**20**). The selected districts were: Nandurbar (low HDI) from Northern Maharashtra (Khandesh), Amravati (Moderate HDI) from Vidarbha region, and Satara (higher HDI) from Western Maharashtra region.

In these districts, we conducted a survey of public health facilities and community-level survey of recently-delivering women.

#### Collection of health facility-level data

We studied 44 secondary- and tertiary-care centres—3 district hospitals (250-bedded), 8 subdistrict hospitals (50 to 100-bedded), and 33 CHCs in the three selected districts. Observations and record reviews were used for collecting data. We surveyed each facility, using a standard form based on the facility survey for EmOC, designed by the Averting Maternal Death and Disability programme (21). Team members with medical background conducted the survey. The data collection from health facilities was undertaken from 9 December 2009 to 31 August 2010. Each facility was visited once without prior notice.

Interviews were conducted with obstetricians (n=15), medical superintendents (n=20), district health officials (n=2), and programme managers (n=2) in the government facilities. We also interviewed obstetricians in the private sector (popular ones whose names were frequently mentioned by mothers) working in or near the towns with CHCs. However, keeping up with the scope of this paper, only those private obstetricians who were contracted-in at the public facilities to provide emergency obstetric care, including care for eclampsia, were included in the study.

#### Collection of community-level data

We conducted the survey in two purposefully-selected blocks in each district. From each block, we randomly selected 10 sub-health centres (SHCs) that are primary-care facilities serving about 5,000 population in plain areas and 3,000 in hilly areas run by Auxillary Nurse Midwifes (ANMs). In this survey, we included 272 villages served by these SHCs. We collected the community-level data in two phases: a birth survey followed by semi-structured interviews.

We conducted the birth survey as part of our study on birth complications and the *Janani Suraksha Yojana* (JSY)—a flagship scheme of the National Rural Health Mission that aims to reduce maternal mortality and morbidity. We used the criteria of the JSY for selecting women for the survey, viz. women above 19 years of age from below poverty line (BPL)/scheduled caste (SC)/schedule tribe (ST) communities, with up to two livebirths. We defined a ‘recent delivery’ as one that occurred within the most recent year prior to the survey and specified it as the period between two major annual festivals in between 1 November 2008 and 31 October 2009.

The identification of households in the communities where a delivery occurred recently was coupled through key informant interviews to a ‘snow balling’ technique to identify additional households. We also used service-records at SHCs and *Anganwadis* to obtain a complete list of potential respondents. We approached the listed households for recruitment of respondents.

We, thus, reached 1,833 women in the three selected districts. This paper focuses on treatment for eclampsia, and only the relevant data are reported ignoring a lot of others analyzed elsewhere.

**T1able 1.** Sampling details for community-level survey

Unit	Recruitment criteria and size	Sample-size
District	HDI and geographic representation	3
Block	Purposive—availability of local support for field work 2 blocks per district	6
SHC	Randomly—10 per block	60
Village	Automatically—all villages covered by selected SHCs	272
Household	BPL/SC/ST households listed through key informant interviews and service-records	-
Survey respondents	All recently-delivering women from BPL/SC/ST families, with up to two livebirths	1,833
Interview respondents	Survey respondents reporting a pregnancy/childbirth-related complication—selection by maximum variation sampling	120
	Number of respondents who reported eclampsia	10

The survey questionnaire aimed at identifying the occurrence of obstetric complications in women. It was validated by five senior obstetricians and two public health experts. The question for identification of eclampsia was: “Did you ever have a convulsion during your recent pregnancy, delivery, or within two days postpartum?” A positive response was further probed to find out: (a) whether such episode occurred for the first time during pregnancy/around delivery, (b) whether there was an absence of a history of epilepsy, and (c) whether there was an absence of fever associated with the convulsion. A positive response to each of these questions indicated a level of eclampsia.

Trained investigators conducted the survey during November-December 2009. Field supervisors verified 10% of the survey forms.

The survey identified women who had one or more obstetric complications (466 of 1,833) and, of them, we selected 120 women for semi-structured interviews for the JSY components of the study, using maximum variation sampling based on (a) receipt of JSY assistance and (b) place and type of delivery. Ten of these interviewed mothers had experienced eclampsia. This paper specifically reports the eclampsia care-related experiences shared by these 10 mothers. Two trained female researchers conducted these interviews during December 2009-March 2010.

The recruitment criteria and sample-size for community-level enquiry are summarized in Table 1.

The study followed three links of investigation: (a) facility-level survey, (b) interviews with obstetricians, and (c) a community-level enquiry into experiences of treatment for eclampsia.

Informed written consent was obtained from all study participants. All interviews were audiotaped and transcribed verbatim for analysis.

Quantitative data were entered and analyzed in Excel. Qualitative data were coded deductively and analyzed for themes and subthemes.

### Ethical approval

Ethical approval for the study was granted by the Institutional Ethics Committee of the Foundation.

## RESULTS

The first section presents findings from the health facility survey, followed by results of the community-level survey.

### Health facility survey

Table 2 presents the performance of the facilities in eclampsia management. Of the 44 facilities surveyed, 32 (73%, 95% CI 60-86) had not administered anticonvulsants to treat eclampsia in the recent three months prior to the survey. Respondents of 37 facilities mentioned that they were equipped to provide MgSO_4_ treatment for eclampsia but all did not have MgSO_4_ readily available.

**T1able 2.** Performance of public facilities in Maharashtra with regard to eclampsia management

District	Facilities surveyed	Facilities that treated eclampsia in recent three months (%)	Facilities where providers mentioned readiness to administer intravenous MgSO_4_ treatment for eclampsia (%)
Satara	18	5	16
Amravati	14	4	13
Nandurbar	12	3	8
Total	44	12 (27)	37 (84)

Source: Health Facility Survey

It was informed that the usual practice is to refer eclampsia patients directly to a higher centre without first stabilizing the patient or giving first-line treatment for lack of readily available MgSO_4_. A private obstetrician who had been hired by a CHC to provide specialty services stressed the need for adequate drug supplies and equipment. While recalling referrals of eclampsia patients she said:

The supply of medicines should be proper. For example, although I can handle a case of eclampsia, I had to refer her because there was no MgSO_4_. It isn't very costly….could be purchased locally but there could be other complications subsequently, and more drugs could be required. There are other things also: if she gets convulsion, immediate suction should be done.

Our attempts to trace referral records at these facilities were not successful due to the unavailability of proper records concerning provisional diagnoses or reasons for referral.

The obstetricians interviewed—both public and private—were aware and mentioned their knowledge of the use of MgSO_4_ as the gold standard for treating eclampsia. However, their responses regarding its use in the management of severe pre-eclampsia (to prevent eclampsia) were unclear.

The reason mentioned by medical superintendents at the government hospitals for not offering MgSO_4_ treatment was the hesitation of obstetricians in handling critical cases which they perceived as ‘taking risks in public service’. They also mentioned that doctors were not confident in administering MgSO_4_ for eclampsia fearing adverse outcomes. However, all obstetricians and medical superintendents interviewed were aware of the routine use of MgSO_4_ for management of eclampsia by private care providers in the vicinity.

### Availability of magnesium sulphate

According to the Indian Public Health Standards (IPHS) guidelines (22), a CHC is expected to have a minimum stock of 50 ampoules of injectable MgSO_4_. These quantities for subdistrict hospitals have not been specified but would logically be more than that for CHCs commensurate to the number of beds at the facility.

The survey listed the number of ampoules of Injection MgSO_4_ available at the facility on the day of the visit. These were physically verified by the investigators. In the case of unavailability, the last day when it was available as mentioned in the stock registers was recorded to determine the duration of the episode of out-of-stock position.

Based on the 50-ampoules criteria, facilities were classified into three categories: (i) those with a stock-out position i.e. zero stock, (ii) those having less than 50 ampoules, and (iii) those with fifty or more ampoules available. The findings are presented in Table 3.

**T1able 3.** Availability of Injection Magnesium Sulphate in public health facilities

District	Health facilities surveyed	Facilities with nil stock N (%)	Facilities with less than minimum expected stock—50 amps N (%)		Facilities with ≥50 amps N (%)
			< 20 amps	≥20 amps	
Satara	18	12 (66)	4 (22)	1 (6)	1 (6)
Amravati	14	11 (79)	0	1 (7)	2 (14)
Nandurbar	12	4 (33)	1 (8)	2 (17)	5 (33)
Total	44	27 (61.4)	5 (11.4)	4 (9)	8 (18.2)

Source: Health Facility Survey

The number of ampoules of the injection available at facilities with less than expected minimum stock ranged from a single ampoule available at one facility to a maximum of 35 ampoules at another. As per recommendation (23), the starting dose for a patient with severe pre-eclampsia or eclampsia would be 20 ampoules of Injection MgSO_4_ (500 mg/ampoule) by the minimum requirement intramuscular (IM) only regimen. Of the 9 facilities having less than 50 ampoules, 5 had stocks less than this bare minimum starting dose for a single patient. This translates to 32 (73%) facilities (95% CI 60-86) in the study districts unprepared in terms of having enough MgSO_4_ to provide the first response to a life-threatening pregnancy complication.

All the 3 district hospitals stored more than 50 ampoules of the injection. Three of the 8 subdistrict hospitals had no stock, and an equal number had less than adequate (50 ampoules) while two had adequate stock. Overall, 10% of the CHCs had the required minimum stock, and all these were in Nandurbar district. No facility in Amravati and Satara district, except the district hospitals, had adequate stocks of MgSO_4_.

Among the three study districts, Satara reported the highest unavailability while the situation in the tribal district of Nandurbar appeared better.

#### Procurement and supply procedure

Drug procurement in the public system of Maharashtra is centralized at the state level under the direction of the public health department, based on a tender system. The secondary-care health facilities receive drugs through stores at the district hospitals. MgSO_4_ is supplied in the standard strength of 250 mg/mL packed in ampoules of 2 mL each for intravenous or intramascular use. Additionally, the facilities are provided funds that can be utilized for local purchase of medicines, if required.

**T1able 4.** Findings from community-level survey

District	Villages surveyed	Recently-delivering women (JSY eligible)	Women reporting eclampsia N (%)
Satara	125	218	4 (1.8)
Amravati	95	803	32 (3.9)
Nandurbar	52	812	23 (2.8)
Total	272	1,833	59 (3.2)

Source: Primary data from community-level survey

#### Reasons for unavailability

The period during which the stock-out position existed at these facilities ranged from 3 months to 3 years. In our quest to understand the reasons for the lack of this drug, almost all medical superintendents reported no supplies from the district headquarter despite the usual requisition through indent forms. Invariably all respondents said that they could procure the drug from the untied funds provided under the NRHM or from facility-strengthening funds for the IPHS. However, no such local purchase of MgSO_4_ was undertaken, except at one facility. The explanation that they could do so if any such emergency case arrived, points to the poor emergency preparedness at facilities.

### Community-level survey

#### Incidence of eclampsia

The survey yielded a high response rate with no refusals of participation by the respondents. We identified 1,833 recently-delivering women from BPL/SC/ST, with up to two livebirths. Our survey showed that 3.2% (95% CI 2.3-4) of the respondents reported a convulsion related to eclampsia during their recent pregnancy or postpartum period (Table 4).

#### Characteristics of mothers with eclampsia

Most mothers reporting eclampsia were less than 25 years old, nearly one-fourth were illiterate, and nearly one-third delivered at home (Table 5).

**T1able 5.** Characteristics of eclampsia cases

Characteristics	Frequency	Percentage
Age		
<18 yrs	0	0
18-25 yrs	49	83
>25 yrs	10	17
Total	59	100
Education		
Illiterate	14	24
1st-4th class	5	8
5th-12th class	37	63
>12th class	3	5
Total	59	100
Place of delivery		
Home	17	29
Public facility	28	47
Private facility	14	24
Total	59	100
Type of delivery		
Vaginal delivery	32	54
Vaginal delivery with episiotomy	18	31
Assisted vaginal delivery with forceps	2	3
Caesarean section	7	12
Total	59	100

Source: Primary data from community-level survey

#### Treatment-seeking and referral for eclampsia care

Table 6 summarizes the first facility mothers approached for eclampsia care and the facilities where such care was received. It is evident that women from even the poorest communities sought care from private hospitals in the event of eclampsia. Also, among those who approached a public facility, some women did not receive treatment for eclampsia there. Some women were referred to a higher public facility but they chose to go to a private hospital while some had self-referrals to private facilities. Whatever the reason for and pattern of referral, it is noted that the facilities that treated women with eclampsia were exclusively in the private sector.

**T1able 6.** Care-seeking by women with eclampsia

District	First contact for eclampsia care			Place of eclampsia treatment		No medical care
	Private	Public	Faith healer	Private	Public	
Satara	1	1	0	2	0	0
Amravati	2	1	0	3	0	0
Nandurbar	1	2	2	2	0	3
Total	4	4	2	7	0	3

Source: Data from interviews with women

Women who went to private hospitals to seek care chose to do so because of unavailability of this treatment at the government hospitals nearby. One of them explained:

If facility that I got at xxx (pvt) hospital was available at the government hospital, why would I have spent so much money; I would have gone to government hospital only.

Women expressed disappointment at seeking treatment for pre-eclampsia and eclampsia at the government facilities in rural areas. A respondent from the tribal district of Nandurbar reported that she went to the PHC, with swelling on hands, feet, and face and headache during pregnancy but got no relief after consuming the tablets received. She recollects having developed convulsions in the following week and was unconscious when family members took her to a private hospital where she was given injections and recovered. She shared:

We took small loans for taking treatment during my pregnancy because when we went to government hospital, their treatment was of no use, so we went to private, that was expensive but was good. The private doctor told me that if I had got proper treatment when I had swelling and headache, I would not have got the condition.

#### Costs of eclampsia care

Traditionally in India, women go to maternal homes for delivery, and parents bear the pregnancy-related expenses. Most of the interviewed women mentioned difficulties the family faced in arranging their eclampsia treatment. One recollected her parents taking a loan of Rupees 8,000 (USD 174) at an interest rate of 10% a month while her mother mortgaged her golden necklace for Rupees 3,000 (USD 65).

#### Awareness and perceptions of eclampsia symptoms

While all respondents with eclampsia had received some antenatal care, none was told of the danger-signs and where to seek care from.

Some women mentioned that swelling on body during pregnancy is considered a normal sign in late pregnancy, and even older women in their families did not think it required attention. Often women also accessed faith healers in addition to medication to calm the ‘evil eye’ that, they believed, caused the convulsion.

## DISCUSSION

To the best of our knowledge, this is the first study of its kind reporting availability of treatment for eclampsia in public facilities in rural India.

Although our study is limited to the public sector and the situation in the private sector is likely to be quite different from our findings, we purposefully focus on public availability of MgSO_4_ treatment in recognition that poor women face financial barriers to access care from private facilities, which makes them more likely to forgo treatment. As this paper reports eclampsia care-related findings from a study of the JSY and of birth complications, the sampling of mothers had to be based on the JSY criteria and, hence, could not be using ideal methods as would an epidemiological study have. Our findings need to be interpreted with this in mind and the purpose of the paper being to draw on the system issues in providing standard treatment for eclampsia in resource-poor rural settings.

Our finding of a 3.2% incidence of eclampsia is comparable with the other reports of an incidence of 1-5% in developing countries. A study from four Indian states reported occurrence of convulsion in pregnant women ranging from 3.8% in Kerala, 4.9% in Andhra Pradesh, 15% in Madhya Pradesh to 20.7% in Bihar (24). Our findings are obtained from women with lower parity, who are at a greater risk of developing eclampsia (25). Self-reported morbidity tends to be higher than the clinically-documented morbidity, and this could be seen as a limitation of our study. However, by using a clear differentiation from convulsions unrelated to pregnancy, we have minimized this limitation. Also, since our findings are reported from recent deliveries and since we asked about major events (convulsions), loss to history is unlikely. Our findings are important because they are based on primary data from our community-level survey. We did not have to rely on secondary statistics from institutions with poor-quality medical records and in areas with sizeable numbers of home deliveries.

### Gap in policy practice for the use of MgSO_4_

The Indian national list of essential medicines (17) codes MgSO_4_ as a level-T drug, signifying its use only in tertiary-level facilities. However, guidelines for the availability of basic EmOC at CHCs and PHCs in the country include management of eclampsia and the use of MgSO_4_ (23). The nurses and midwifes working at PHCs/CHCs are trained to be skilled birth attendants who are authorized to administer MgSO_4_. Such ambiguities in the authorization to administer MgSO_4_ may be one of the factors influencing its use or unuse at lower levels of care. In contrast, WHO recognizes that MgSO_4_ administration and monitoring are relatively straightforward without the need for expensive equipment. WHO further recognizes that IM administration can be used when staff experienced in its IV administration and monitoring are not available. Several resource-poor countries report the use of MgSO_4_ at primary-care facilities (26).

### Poor inventory management

Our findings indicate poor inventory control. The present practices in managing stocks seem to be ineffective in curbing stock-outs. The state public system should replace the older supply systems to better utilize technology and practices that have proven to be effective. For example, the drug procurement in Tamil Nadu state is reported to be efficient and is decentralized under the management of the Tamil Nadu Medical Services Corporation (27).

### Poor monitoring processes

Our study shows serious inadequacies in the availability of MgSO_4_ in the CHCs in Maharashtra. This contrasts with the findings of the Common Review Mission (CRM) that surveyed health facilities to assess the progress of the National Rural Health Mission (NRHM) and reported “drugs and supplies are in abundance in the subcentres, PHCs, and in CHCs” (18). The Mission did not make a specific mention of the availability of MgSO_4_, although it is an important drug to treat maternal complications, especially in the light of the NRHM's goal of reducing maternal mortality. Similarly, the proforma for reporting gaps for the IPHS (28) includes Oxytocin and Ergometrine (other important emergency obstetric drugs) but not MgSO_4_. This leads to a neglect in maintaining the supply of MgSO_4_. The argument is that MgSO_4_ does not cost much and that pre-eclampsia or eclampsia affects a smaller proportion of the population compared to other diseases. This draws attention to the need to align process assessments with specific programme objectives and to emergency obstetric care needs.

### MgSO_4_ versus other drugs for eclampsia

Our finding that care providers consider the availability of diazepam and phenytoin—other drugs for treatment of convulsions—bridges the gap of MgSO_4_ availability is a matter of concern as it is known that these drugs are not indicated for treatment of severe pre-eclampsia, unlike MgSO_4_ (29), and that appropriate use of MgSO_4_ in pre-eclampsia can prevent eclampsia. Notably, both pre-eclampsia and eclampsia are serious disorders and can be life-threatening.

### Emergency obstetric care preparedness

National Rural Health Mission (NRHM) provides funds and authority to medical superintendents to purchase drugs locally. However, this seems to have become an accepted excuse for every shortage. This strategy does not work. Our study shows that it can result in significant (and harmful) delays in care when facilities rely on acquiring MgSO_4_ only after the patient arrives. Delay in initiating treatment is known to cause several maternal deaths in India (30). Delay in the diagnosis and treatment of severe pre-eclampsia and eclampsia can result in permanent damage to the brain tissues and other vital organs. The delays in care lead to costly critical care for the mother and the newborn and result in long-term problems in the premature or intrauterine growth-retarded baby. The present study shows that there is a need for an urgent correction of inventory management in resource-poor Indian settings to allow adequate and sustained availability of these medicines in health facilities.

The findings about low confidence among public sector specialists to administer MgSO_4_ treatment for eclampsia draw attention to move a step forward from not only placing qualified personnel in facilities but also ensuring their competence in delivering the desired services.

### Birth preparedness and complication readiness of mothers

Our findings of poor birth preparedness and readiness of mothers in being unaware of the symptoms of eclampsia and of treatment facilities in the vicinity, inspite of having received some ANC, indicate the missed opportunity to educate mothers at the time of ANC. Not merely the quantum, as it appears from current practices, but also the quality of contents of ANC services is an equally important factor that needs attention from the managerial and policy levels. An informed family and mother able to identify danger-signs of eclampsia can reduce delays in seeking appropriate care and avoid the medical and economic consequences thereof. For example, a study in Jamaican setting showed that the use of simpler methods, like pictorial card, increased mothers' awareness and improved response to the symptoms of eclampsia, reduced the incidence of eclampsia and aided the communication between health workers and mothers (31).

### Recommendations

Our findings draw attention to the need for clear guidelines for eclampsia management with no ambiguities, and that are in consonance with related orders from other concerned departments.

An urgent requirement is the need to develop updated inventory control methods to improve pharmaceutical logistics in the health system. We recommend that policy-makers provide the necessary resources and systems for improved logistics performance. In addition, training is required for mid-level managers responsible for supervising the logistics process and for personnel involved in implementing the specific changes required.

Finally, we urge for an approach of providing extended and quality training to care providers so that they are confident to initiate treatment for pre-eclampsia and eclampsia with MgSO_4_ even in primary- and secondary-care facilities. Equally important is the complementary training, with sustained supplies, like pre-filled eclampsia treatment kits.

Overall, our findings re-emphasize the need for facilities to do a better job of preparing for complicated births in addition to teaching women to recognize the signs of pre-eclampsia and eclampsia when seeking medical care.

## ACKNOWLEDGEMENTS

The authors gratefully acknowledge the State Health Systems Resource Centre, Maharashtra, for support to this study. We obtained formal support to the study from the State Health Systems Resource Centre, National Rural Health Mission (NRHM) Division, Government of Maharashtra. Dr. Dileep Mavalankar, Dr. P.P. Doke, and Dr. C.A.K. Yesudian for technical advice; Ms Sunita Mane and Ms Ashwini Jadhav for research assistance; Shramjivi Janata Sahayak Mandal, Satara, Janarth Adivasi Vikas Sanstha, Shahda, Nandurbar and Department of Community Medicine, Datta Meghe Institute of Medical Sciences, Wardha, are also acknowledged for supporting the birth survey; Peter House for English language editing of this manuscript and The John D. and Catherine T. MacArthur Foundation, USA, for financial support.
